# Unraveling the Interconnection Patterns Across Lung Microbiome, Respiratory Diseases, and COVID-19

**DOI:** 10.3389/fcimb.2020.619075

**Published:** 2021-01-28

**Authors:** Elisavet Stavropoulou, Konstantia Kantartzi, Christina Tsigalou, Theocharis Konstantinidis, Chrissoula Voidarou, Theodoros Konstantinidis, Eugenia Bezirtzoglou

**Affiliations:** ^1^ CHUV (Centre HospitalierUniversitaire Vaudois), Lausanne, Switzerland; ^2^ Department of Infectious Diseases, Central Institute, Valais Hospital, Sion, Switzerland; ^3^ Nephrology Clinic, Department of Medicine, Democritus University of Thrace, Alexandroupolis, Greece; ^4^ Laboratory of Microbiology, Department of Medicine, Democritus University of Thrace, Alexandroupolis, Greece; ^5^ Department of Public Health P.U., Arta, Greece; ^6^ Laboratory of Hygiene and Environmental Protection, Department of Medicine, Democritus University of Thrace, Alexandroupolis, Greece

**Keywords:** lung microbiome, lung-gut axis, lung in health and disease, lung, microbiota, COVID-19, SARS-CoV-2, lung immunity

## Abstract

Albeit the lungs were thought to be sterile, recent scientific data reported a microbial microbiota in the lungs of healthy individuals. Apparently, new developments in technological approachesincluding genome sequencing methodologies contributed in the identification of the microbiota and shed light on the role of the gut and lung microbiomes in the development of respiratory diseases. Moreover, knowledge of the human microbiome in health may act as a tool for evaluating characteristic shifts in the case of disease. This review paper discusses the development of respiratory disease linked to the intestinal dysbiosis which influences the lung immunity and microbiome. The gastrointestinal–lung dialogue provides interesting aspects in the pathogenesis of the respiratory diseases. Lastly, we were further interested on the role of this interconnection in the progression and physiopathology of newly emergedCOVID-19.

## Introduction

The function and structure of the microbial communities are determined by complex microbial interconnections and network patterns of unique microbiomes. The microbiome is a community of microorganisms colonizing a particular micro-environment in or out of the human body. Additionally, they participate actively in the metabolism and establish potent positive or negative interactions and relationships such as synergism, commensalism, parasitism, antagonism, and other which could explain to some extent the genetic diversity of microbial populations. Yet, following mutations or selective pressure functional microbial genes alter their functionality in the environment (Cordero and Polz, 2014), and via adaptive genes can increase colonizing ability (Hughes et al., 2008). Meanwhile, it was shown that phylogenetically close taxa were more frequently found in the same microenvironment (Chaffron et al., 2010).

Without any doubt, unraveling and exploring the involved microbial patterns and getting a better knowledge of the microbiota profile should clarify the role of the microbiome in health and disease and should lead to the development of more effective or even alternative therapeutic strategies. Outbreaks of various diseases seem to be more common, aggressive, and dangerous during the last years due to the changing environment and the climate warming. New pathogenic bacteria appeared and more than 60% of them originated from animals. Moreover, changes in land and agriculture practices and deforestation alter the environment. Mosquitoes, ticks, and other vector-borne insects are in a rise in the ‘previously’ mild climate European countries due to the shifting climate patterns which lead species to change their habitats. “Forgotten diseases” such as yaws and anthrax showed an emergence. For example, the *Nipah* virus causing severe encephalitis crossed the species border and passed from animal farms to humans in Malaysia in 1999 (Ang et al., 2018). On the other hand, calamitous rainfalls following climate shifts skyrocket rodent population and an outbreak of pulmonary syndrome caused by *Hantavirus* emerged in Panama in 1999 (Bayard et al., 2004). It is believed that more than 3,200 coronaviruses species are found in animal reservoirs such as bats and birds awaiting the favorable time to cross the species level and then pass to the humans (Shuman, 2010). Coronaviruses present a large range of disease states extending from simple flu-like illness and gastrointestinal disease to a severe acute respiratory syndrome (SARS). The novel coronavirus has spread rapidly to multiple countries and has been declared a pandemic by the World Health Organization showing the ‘terrifying awake of the nature’ over human civilization and politics (WHO). The most common symptoms of COVID-19 disease comprise mild fever, dry cough, fatigue, anosmia, sore throat, and diarrhea (Ferreira-Santos et al., 2020). However, there are an important number of disease carriers that silently spread the disease. Lung infection observed following SARS-CoV-2 virus invasion could finally lead to death, and the main complications include pneumonia, acute respiratory failure, acute respiratory distress syndrome, acute kidney damage complicated with acute liver damage and septic shock (Huang et al., 2020).

As stated, the COVID-19 infection caused specifically a broad spectrum of severe respiratory diseases and uncertainly the lung microbiome may play an important role to the development of the disease (Yuki et al., 2020). Data used for the present review were identified by a Medline database of systematic reviews and peer-review articles published in English since 1997. However, most articles concerning the main developed concept were recent publications. Articles that were consulted are included in the list of references if they presented an original approach. The main keywords used were: Gut, Lung, Microbiome, Lung microbiome, Gut–Lung axis, Lung in Health and Disease. Internet sites of paramount interest based mostly on the above keywords were identified such as WHO, NIH, Wiley on line library, Science Direct. Finally, important references mentioned in the selected articles were also explored and taken into account.

The subject of this review is divided by subheadings in order to focus in the different questions and thus to provide a concise and precise description of the current knowledge.

## The Lung Microbiome in Health

In order to improve understanding of the microbial flora involved in human health and disease, scientists proceeded to the characterization of the microbial communities of healthy individuals, across different body sites on the human body: nasal passages, oral cavity, skin, gastrointestinal tract, and urogenital tract. New technologies and specifically the 16S rRNA sequencing was applied to identify the complexity of microbial communities in the human body (NIH Human Microbiome Project - Home). The metagenomics Whole Genome Shotgun (WGS) sequencing brought to light the complexity of the human pathways and functions in relation to the human microbiome.

As known the most studied microbiome is the gut microbiome. Little was known on the lung microbiome which was considered as sterile in multiple studies and textbooks going back often without citations (Cox et al., 2019). The human newborn is deprived of bacteria before birth. The establishment of normal microflora is a continuous and complex pattern which initiates at delivery and goes on through consecutive stages under the influence of external and inner factors (Adlerberth, 2009; Bezirtzoglou and Stavropoulou, 2011; Arumugam et al., 2011). During vaginal delivery, bacteria of maternal origin colonize the newborn oral cavity (Lif Holgerson et al., 2011). Certainly, oral bacteria enrich and shape the lung microbiome (Segal et al., 2016). It was observed that in healthy people, bacterial DNA of several oral bacterial species, such as *Prevotella* and *Veillonella* was found in the lower respiratory tract owing to the topographical continuity (Charlson et al., 2011) as the oropharynx and the tracheobronchial tree are adjacent and continuous to the oral cavity (Mammen et al., 2000). Moreover, the exposure pattern during the neonatal period influence the microbial colonization and species variation (Bezirtzoglou, 1997; Rutayisire et al., 2016). The dominant genus found in lung are *Prevotella, Veillonella*, Streptococcus (Dickson et al., 2016a), *Pseudomonas, Fusobacteria* and rarely *Haemophilus* and *Neisseria* (Beck et al., 2012). These genera are easily colonizing the oxygen rich, damp ciliated mucosa of the larynx and the tracheobronchial tree in continuity to the oral purlieu. Yet, due to the air passage, mucus is continuously enriched with microorganisms. However, the respiratory mucus embedded with lipid-rich surfactants showed bacteriostatic effects against several bacteria and also it seems that there is an eternal fight between bacteria and alveolar macrophages in the lung (Wu et al., 2003).

In terms of phylum, varied phylogenetically microbial populations were found: *Proteobacteria, Firmicutes, Fusobacteria, Actinobacteria* and *Bacteroidetes* (Charlson et al., 2011; Moffatt and Cookson, 2017). Concerning the mycobiome in health individuals, *Eremotheriumsinecaudum, Vanderwaltozymapolyspora, and Systenostrema* alba of the *Saccharomycetaceae* family as well as several terrestrial microsporidia were isolated (Nguyen et al.).

Thus assuming, it is generally accepted actually that there is a lung microbiome in balance with different factors such as microbial penetration and expulsion from the airways and even growth of colonizing bacteria adapted to the local conditions and environment (Dickson et al., 2014).

## Lung Immunity

The lungs are exposed to multiple hazards on a daily base. Virtually, the lung cells play a role in assessing whether they will respond or not. Originally, the upper respiratory tract role is dedicated to preventing entry of noxious particles. When sizeable particles deposit in the nasopharynx and tonsillar regions, they are cleared mechanically by coughing or sneezing. Besides this, the remaining non-eliminated particles are expelled smoothly *via* rhythmic movements of microscopic cilia to the upper airways (Beutler, 2004).

Antimicrobial peptides (AMPs) LL37 are expressed in the epithelial cells of the upper respiratory tract. AMPs are known to possess an antimicrobial activity against microorganisms either by direct binding to the bacterial surface or binding following microbial opsonization to be recognized by innate immune cells (Wang, 2014). The alveoli are tiny air sacs distributing oxygen within the body. They are located at the terminal branches of the lungs where most of the gaseous exchange occurs. Immune cells are poorly represented in the alveoli, and they are mainly consisting of alveolar macrophages (AMs) which provide the primary phagocytic activity against microorganisms without triggering inflammatory responses (Martin, 2000; Martin and Frevert, 2005).

Except for the upper airspaces part, the lung immune system is presented by the intraepithelial lymphoid tissue of lungs (ILTL). The immune response of ILTL in all stages is orchestrated by dendritic cells (DCs). Furthermore, the triad of DCs, lung microbiome and AM are actively participating in the bacterial recognition. T cells (predominantly cytotoxic), B lymphocytes, neutrophils, and rarely mast cells are observed in ILTL (Freeman and Curtis, 2017).

As known, epithelial cells are also involved in lung immunity by secretion of chemokines, cytokines, and antimicrobial compounds (Lacy, 2015). During dysbiosis, AM phagocytes adhere to the opsonins in order to facilitate phagocytosis as opsonized encapsulated bacteria should be ingested with more difficulty (Gordon and Rice, 1988; Arango Duque and Descoteaux, 2014; Murray and Stow, 2014). Additionally, immature DCs migrate into tiny airways, where after activation by AM, promote a pro-inflammatory microenvironment (Kawamura et al., 2005; van Rijt et al., 2011; Shaykhiev and Crystal, 2013).

Neutrophils and macrophages are involved in the innate immunity as first line of non-specific defense against pathogens. Their role is to engulf and destroy the pathogenic bacteria. Neutrophils’ recruitment and activation are observed in response to inflammation status. However, macrophages exhibit decreased phagocytosis in the large lung airways during the progression of chronic obstructive pulmonary disease (COPD) (Bu et al., 2020). Dropping of the phagocytic capacity of macrophages impair the lung function and produce a chronic inflammation status (Bu et al., 2020).

**Figure 1:**
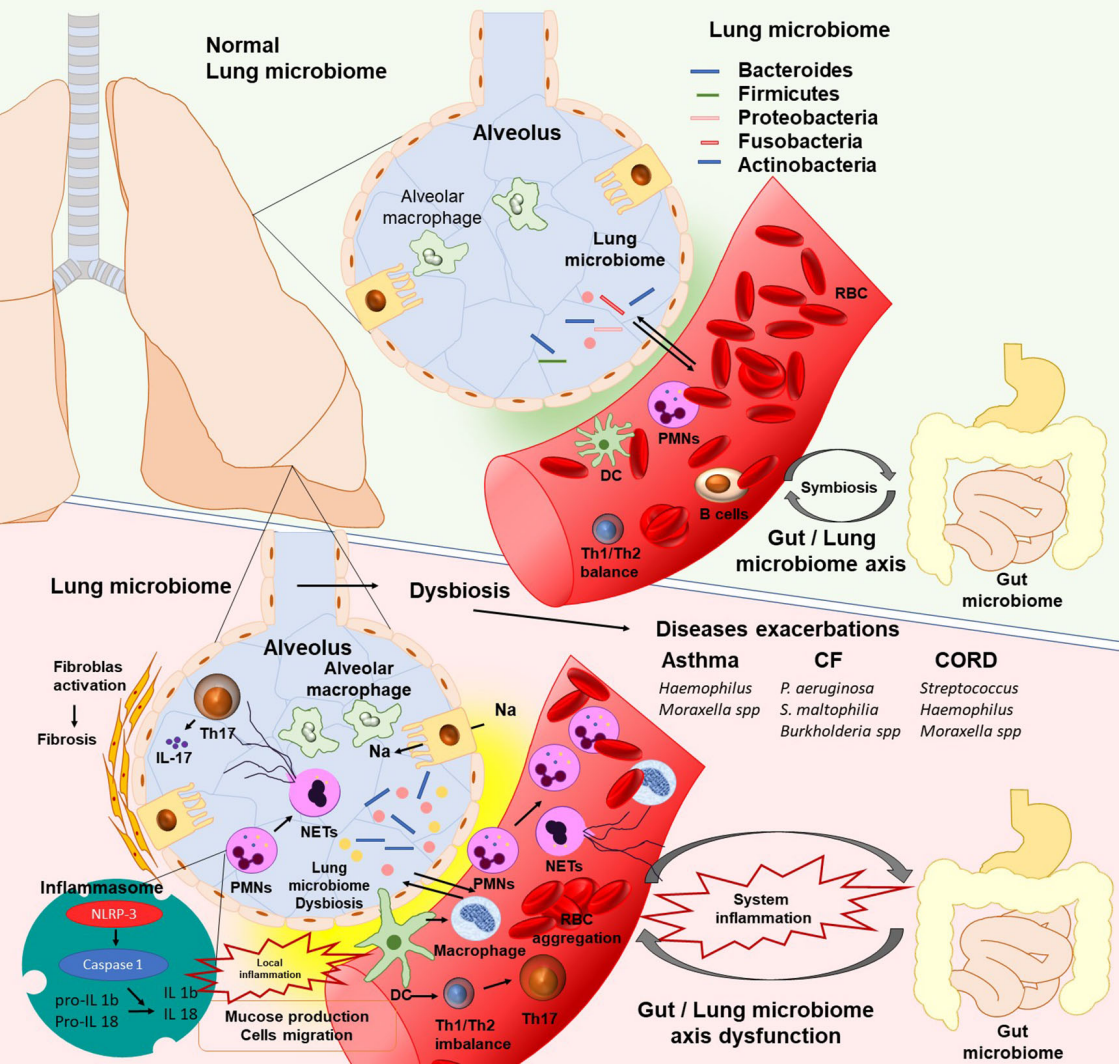
The existence of lung microbiota plays a critical role in lung homeostasis. In human’s lungs the bacterial load is increased from neonates to adulthood and contain Proteobacteria, Firmicutes, Fusobacteria, Bacteroidetes, and Actinobacteria bacterial phyla. Lung microbiota, in the same manner as gut microbiota, can promote the polarization of naïve T cells in the lungs from Th2 to Th1 to protect against asthma and allergy. Moreover, microbiota promote the differentiation of alveolar macrophages, inhibit the exaggerated inflammatory response, and promote protection against pathogens. The lung microbiome dysbiosis leads to dendritic cells activation and antigen presentation. Immune cells respond to microbial colonization through the pattern recognition receptors (PRRs). Activated cells migrate into the tissue, produce series of cytokines such as IL-1b and IL-8, and release Neutrophil Extracellular Traps (NETs), which contribute to local inflammatory response. Moreover, alterations in cytokines, especially IL 17(NETs or Th17 inducted), promote pathologic fibrotic remodeling (PMNs, polymorphonuclear leukocytes; DC, Dendritic cells; IL, Interleukins; CF, cystic fibrosis; CORD, Chronic obstructive pulmonary disease).

At the same time, in the small airways macrophages demonstrate high pro-inflammatory potential (Shapiro, 1999; Bu et al., 2020) (**Figure 1**). Moreover, Natural killer T (NKT) cells, eosinophils, and mast cells are incriminated to have a complementary function in the inflammatory process of small and mediate alveolar tissue that leads to chronic airway inflammation (O’byrne and Postma, 1999). Likewise, activated neutrophils are able to release Neutrophil Extracellular Traps (NETs) which promote a proteolytic microenvironment through enhanced expression of IL-17 resulting in the fibrotic repair of small airways (Bosch and Ramos-Casals, 2014; Chrysanthopoulou et al., 2014).

Epithelial cells seems to play a crucial role in the initiation of the immune response as they recognize viral antigens through cytoplasmic Pattern Recognition Receptors PRR stimulation, for instance Toll like receptors (TLRs) and intracellular sensors, including Retinoic acid-Inducible Gene-I-like receptors(RIG-I-like receptors) (Xiang and Fan, 2010; Whitsett and Alenghat, 2015). As a result, coupling induces a signaling avalanche effecting in the upregulation of type I and III interferons and initiating the inflammatory response and the differentiation of adaptive immunity involved cells (Xiang and Fan, 2010; Whitsett and Alenghat, 2015). Alveolar macrophages and DCs contribute to the secretion of the cytokines and chemokines (Kolli et al., 2014). Finally, PRR coupling and cytokine secretion induce DC maturation and adaptive immune response (Kranzer et al., 2004).

The adaptive immunity response is specific to the pathogen by clonal expansion of T and B lymphocytes and consists of a second line defense (Adams et al., 2020). This effect is highly specific and long maintained by memory T cells. Affinity maturation is expressed on TCRs found on T lymphocytes leading to a more specific antigen binding capacity (Zhu et al., 2015).

T lymphocytes carry the CD4 or CD8 receptors (Gao et al., 2020). The CD4+ T cells are known as T helper (Th) cells due to their capacity *via* cytokine production to stimulate immune cells (Zhu et al., 2015). Yet, the CD4 regulatory T cells (Tregs) are able to regulate immune feedback. Particularly, Tregs *via* the production of IL-10 suppress T cell differentiation (Ding et al., 2012). The CD8 T cell receptors are known as cytotoxic T cells due to their ability to produce molecules and cytokines (Altmann, 2018). This occurs in the lymph nodes of lung parenchyma, bronchioles, and trachea. CD4 T cells are divided in subsets following the secreted cytokines [T helper type 1 (Th1) cells and T helper type 2 (Th2) cells] which showed different functions (Gao et al., 2020). These cytokine production ends to a consistent local host protective response. However, thereafter most CD4+ T cells die (necrosis, apoptosis), and few CD+ cells are present as memory cells in the host (Maly and Schirmer, 2015).

Concerning the B cells’ specific response which is taking place in lymphoid tissues such as lymph nodes, mucosa-associated lymphoid tissue of broches and nose, it seems to promote IgA (Ladjemi et al., 2015). Affinity maturation is not usually expressed as B cells are short-lived (Zhu et al., 2015). The basic differentiation process of B cell is dependent on T cells and outcomes in the high-affinity antibody cells and memory B cells (Schuurman and Quesniaux, 1999; Zhu et al., 2015). Specifically, mature DCs fitting out viral antigens translocate to lymphoid tissues (Tang et al., 2010). These antigens are recognized, and differentiation proliferation processes are initiated (Tang et al., 2010). Specifically, antigen recognition is achieved through the CD4+ Th cells presented by the B cell *via* major histocompatibility complex class II and alternate Immunoglobulins isotype classes (IgM to IgG and IgA) (Couture et al., 2019). At this stage of the process, several B cells participate in the Germinal Center (GC) followed by hyper-mutation and affinity maturation (Anderson et al., 2009; Zhu et al., 2015). In this vein, affinity maturation is stimulated by CD4+ follicular helper T cells (TFH cells) (Zhu et al., 2015). TFH cells possess a pool of markers, such as chemokine receptor CXCR5, ICOS, CD28, PD-1, CD40L, and SAP as well as their driven specifying transcription factor Bcl-6 (Suh, 2015). TFH cells are found in GCs and DCs, and this permits their strait relation to B cells (Chen et al., 2012; Couture et al., 2019). TFH cells’ signals induce ulterior selective differentiation of B cells to low-affinity suboptimal clones (Chen et al., 2012; Suh, 2015). As stated TFH cells express multiple markers, shift immunoglobulins isotype classes to IgA thus stimulating Bcl-6 expression in B cells, and resulting in the memory generation (Suh, 2015; Zhu et al., 2015). Those TFH cells are responsible for a durable protective antibody response and in this way should be of paramount importance in the vaccines design (Suh, 2015; Zhu et al., 2015). Therefore, the cells TFHs role are not fully investigated. Recent research investigates novel ways as the microRNA cluster miR-17–92, for TFHs’ function, differentiation and migration to B cells (Masopust and Schenkel, 2013; Chiu and Openshaw, 2015). Moreover, it seems that microRNA cluster miR-17–92 inactivation impedes their functional role and the production of high affinity antibodies (Sakai et al., 2017). Without any doubt, investigation of T cell-specific expression of the microRNA cluster miR-17–92 and its importance on TFHs functional role should be promising in the development of effective vaccines (Baumjohann et al., 2013; Skinner et al., 2014).

## The Gut–Lung Microbiome Axis

The gut–lung axis (GLA) consists of a bidirectional communication between the two organs (Enaud et al., 2020). Modulation of the intestinal microbiota may cause lung disease. Gut microbes are recognized by the cells of the immune host by inducing the production of cytokines. After lung inflammation, the bloodstream is invaded by metabolites, immune signals, bacteria, and bacterial products that interact between the lungs and the intestine. In fact, the gut–lung axis concept implicates not only host–microbe interactions but also microbe–microbe interactions (Enaud et al., 2020). From an aspect, the gut–lung intercommunication maintains the host homeostasis in healthy individuals (Enaud et al., 2020).

However, most studies investigated the potential of the bacterial microbiota (bacteriobiota); it is of note that there is a fungal microbiota (mycobiota) that remains less studied (Nguyen et al.) as well as a viral component microbiota (virobiota). Viruses seem to be involved frequently in the development of multiple respiratory diseases (Mitchell and Glanville, 2018), and this is the case of the recent pandemic COVID-19 which affects mainly the respiratory system. Recent studies report that there is another communication between these three kingdoms (Enaud et al., 2020). *Pseudomonas aeruginosa* produces volatile sulfur compounds enhancing growth of *Aspergillus fumigatus* (Briard et al., 2019). There is a reciprocal communication between Streptococcus and *Candida* increasing biofilm formation or enhancing *Candida* pathogenicity (Diaz et al., 2012).

While the prevalent bacterial phyla are identical in lung and gut microbiomes, at the species level they are different. It is stated that a balanced intestinal microbiota is crucial for our health as gut microbiota possess an enormous metabolic profile that determines host health (McAleer and Kolls, 2018). Recent studies disclose the association of gut microbiota dysbiosis with multiple disease states (McAleer and Kolls, 2018). The GLA axis consists of a continuous interconnection between gut and lung that allows passage of gut microbial metabolites, endotoxins, cytokines, and hormones through the bloodstream to the lung. However, when lung inflammation occurs, changes in the gut microbiota are observed (Dumas et al., 2018) proving the bidirectional status. Without any doubt, understanding the impact of the gut microbiota on remote organs and systems is of accrued interest. However, it is not clarified yet if it is involved in the etiology or development of the lung disease (Zhang et al., 2020).

As known, antibiotics given in early life cause alterations of the gut communities and predispose to the development of allergy (Russell et al., 2012). The approach of germ-free animals is of high interest as they are bacteriologically sterile. Germ-free mice are extremely susceptible to acute lung infections caused by *Pseudomonas aeruginosa*, *Streptococcus pneumoniae*, and *Klebsiella pneumoniae* (Brown et al., 2017). In this vein, there is evidence that the gut microbiota amplifies the respiratory system defense potential *via* the granulocyte-macrophage colony-stimulating factor (GM-CSF) signaling (Brown et al., 2017) achieving pathogenic bacteria leveling and elimination through extracellular signal-regulated kinase signaling by alveolar macrophages (Brown et al., 2017). Finally, the intestinal microbiota *via* interleukin-17A induces increased pulmonary GM-CSF production in response to infection (Brown et al., 2017).

Nucleotide-binding oligomerization domain-like receptors (NOD-like receptors; NLRs) are known to be involved in the microbiota signals to regulate neutrophil functions (Clarke et al., 2010), inflammatory responses (Hergott et al., 2016) and hematopoiesis (Iwamura et al., 2017). The role of the gut microbiota and the receptor Nod2 in enhancing the mucosal adjuvant activity of Cholera Toxin (CT) was stated (Kim et al., 2016) in germ-free animals. *Staphylococcus sciuri* has proven to possess high Nod2-stimulatory activity. Thus, *Staphylococcus sciuri* mono-associated to germ-free mice produces a powerful Cholera Toxin (CT) activity (Kim et al., 2016).

As stated, NLR pattern recognition receptors can regulate inflammatory response (Hergott et al., 2016; Brown et al., 2017). Notably, the authors found the Nod2-activating recipients of the gut microbiota to adjust the antibacterial immunity of the lung and immune homeostasis (Brown et al., 2017). *M*. *tuberculosis* is the agent of tuberculosis (TB). Recent studies in patients with tuberculosis showed a gut microbiota with predominance of butyrate and propionate-producing bacteria such as *Faecalibacterium*, *Phascolarctobacterium, Eubacterium* and *Roseburia* (Hu et al., 2019a), while a decrease of short-chain fatty acid (SCFA)-producing bacteria was effective (Saitou et al., 2018).Moreover, in the intestine of tuberculosis patients a decrease in amino-acids and vitamins biosynthesis is observed (Saitou et al., 2018).

*Prevotella* and *Lachnospira* were detected in low levels in tuberculosis patients (Luo et al., 2017). However, patients undertaken tuberculosis treatment showed important shifts in their gut microbiota as *Bacteroides fragilis* and *Bacteroides OTU230* numbers were found in increased levels, while the phylum *Firmicutes* and genus *Clostridiales* were considerably reduced (Hu et al., 2019b). Lastly, *Helicobacter pylori* was found to protect lung from *M*. *tuberculosis* infection (Schuijt et al., 2016; Tarashi et al., 2018).

The gut microbiota has also an impact upon *Streptococcus pneumoniae* lung infection (Henriques-Normark and Tuomanen, 2013). Recently, the interconnection between lung and *Staphylocosccus aureus* lung infection was investigated in mast cell-deficient mice. Gut dysbiosis and higher bacterial lung burden were found. Moreover, mast cells’ presence into the lung rebuilts the host protection against *S. aureus* and gut dysbiosis (Liu et al., 2019).

Respiratory viral infections seem to be influenced by the microbiota balance. *Influenzae* virus (IV) and respiratory syncytial virus (RSV) in animals showed a decrease in the phylum *Firmicutes* while *Bacteroidetes* were increased (Groves et al., 2018). *Pneumocystis* pneumonia infection in mice without CD4+ T cell modifies significantly the gut microbiota community (Samuelson et al., 2016).

Gut associated bacteria may enter the lungs across the bowel wall *via* a process called translocation (Mielcarek et al., 2011; Mukherjee and Hanidziar, 2018). The process seems to be enhanced by gut and alveolar permeability (Yacobi et al., 2010). Gut permeability is known to occur during acute intestinal conditions and sepsis (Vaishnavi, 2013). Permeability of the alveoli is observed in Acute Respiratory Distress Syndrome (ARDS) as a result of direct or indirect epithelial injury (Cardinal-Fernández et al., 2017). Critically ill patients’ present increased intestinal and alveolar permeability due eventually to the increased gut–lung translocation (Assimakopoulos et al., 2018). Authors reported (Xu et al., 2019) an interconnection between dysbiosis in critically ill patients and the risk of ARDS development. This interconnection of the gut and lung has been shown in both animal and human studies as discussed (Barcik et al., 2020). Gut-associated lymphoid tissue (GALT) and Inducible bronchus-associated lymphoid tissue (iBALT) seem to have similar morphology and function which is the immune response regulation (Barcik et al., 2020). Their main functions comprise the production and secretion of IgA at the mucosal tissues and cell T helper (Th) and cytotoxic (Tc) responses (Cesta, 2006).

The concept of this interconnection is stated on the fact that shifts in the intestinal microbiome influence lung disease burden and *vice versa.* Studies state the significance of the immune system maturation in early life (Sokolowska et al., 2018). It is also stated that childhood exposure to microorganisms, the so called hygiene hypothesis protects against allergy and disease (Stiemsma et al., 2015). In humans, histamine produced by intestinal bacteria showed important immunoregulatory functions (Smolinska et al., 2014). It is of note that the local immune reaction produced in GALT and iBALT lymphoid tissues can induce systemic immune responses, withal the mucosal immunity might act as a whole for producing immune response (Cesta, 2006). Epithelial cells are involved in lung immunity by secretion of chemokines, cytokines, and antimicrobial compounds (Whitsett and Alenghat, 2015) leading to modification of the immune response at distal sites (Budden et al., 2017). Yet, intestinal immune cells migrate *via* the mesenteric lymph system and blood to the lung where they have enhanced functions (Stubbington et al., 2017). The authors observed increase of the gut bacterial population following stimulation of the mouse lungs with lipopolysaccharide (LPS) (Anand and Mande, 2018). Short Chain Fatty acids (SCFAs) produced by the intestinal microbiome following dietary fiber fermentation by bacteria support immunity function as they effect as signaling molecules on resident antigen-presenting cells to attenuate the inflammatory response in the lungs (Anand and Mande, 2018; Cait, 2018).

They can be found in other sites circulating in the blood and thus they showed inhibitory effects on pro-inflammatory responses in the lungs (Stubbington et al., 2017). Moreover, liver appears to impede the innate immune response produced by SCFA ligation to G protein receptors or even by repressing the mevalonate pathway through HMGCoA reductase (Young and Hopkins, 2017). High amounts of SCFAs such as butyrate and propionate in children intestinal microbiota are less likely to develop lung pathologies (Roduit and on behalf of the PASTURE/EFRAIM study group, 2019). Likewise, administration of SCFAs in animals enhance the transcription factor FOXP3 by inhibiting deacetylation of histone, thus bracing T regulatory cells (Tregs) and production of IL-10 (Arpaia et al., 2013). Similarly, studies mentioned that intestinal metabolites such as oxylipins and biogenic amines have pro-inflammatory and anti-inflammatory potential (Ávila-Román et al., 2016).

Segmented filamentous bacteria (SFBs) in the gut microbiome of animals and humans are participating in the immune regulation (Yin et al., 2013; Hedblom et al., 2018). Specifically, SFBs regulate CD4+ T-cell polarization into the Th17 pathway involved in lung fungal infections response (McAleer et al., 2016; Bradley et al., 2017). Recent studies highlight the role of the gut innate lymphoid cells to be associated to the lungs tissue repair following inflammatory signals upon IL-25  (Liu et al., 2018).

Administration of the probiotic *Bifidobacterium lactis* HN019 (Gill et al., 2001) is correlated to the elevated number of mononuclear leukocytes as well as to an increased phagocytic and lytic activity (Gill et al., 2001). Reversely, in a global study (COPDMAP study) scientists reported that respiratory infections impact upon the intestinal microbiome mediated by Th17 cells (Wang and on behalf of COPDMAP study, 2017). Recently, it is shown that this bidirectional dialogue is not only of concern to the bacterial potential but it is related to fungal one as well (Li X. et al., 2020). The role of resident intestinal macrophages in airway inflammation and fungal dysbiosis is highlighted (Leonardi et al., 2018).

Considering the above, there is an evidence of bidirectional interconnection. Without any doubt gut microbiota holds a key role to regulate host homeostasis and promote resistance to respiratory infections (Brown et al., 2017). However, it is mainly thought to be associated with the PRR basal activity, specific ligands of the microbiota and intestinal dysbiosis (Chu and Mazmanian, 2013); there is a long way for clarifying the involved mechanisms and pathways.

## The Lung Microbiome in Diseases

Each one of us has our own unique microbiota as multiple factors such as diet, environment, ethnicity, hormonal status, hygienic habits which are crucial determinants to its composition (Bezirtzoglou, 1997). When microbial populations are disturbed, a negative impact called dysbiosis is produced. Similarly, higher abundance and species variation are observed in chronic disease states of the respiratory tract. Moreover, there is a shift in microbial populations. It is reported that several fungi associated with the intestinal dysbiosis could enhance the severity of asthma as intestine and lung communicate and work in tandem (Turturice et al., 2017). The study was carried out based on cytokine markers of inflammation (IFN-*γ*, IL-17F, TNF-α and MIP-1*β* and G-CSF) which were increased. Furthermore, the patient microbiome was diversified, and species such as *Actinomyces ondontolyticus, Actinomyces oral taxon 180, Neisseria meningitidis, and Streptococcus pneumoniae* were dominant (Mortaz et al., 2013). It must not also be neglected that antibiotic overuse or misuse could disrupt the microbiota balance (Bezirtzoglou et al., 2008).

*Proteobacteria* including genus of *Haemophilus, Neisseria, Pseudomonas, Rickettsia* and *Moraxella* species were pronounced in asthma and associated usually with the uncontrolled asthma. Surprisingly*, Firmicutes* with the genus *Lactobacillus* were isolated in several asthmatic patients (Park et al., 2014) as well as the genus *Clostridium* in children with airway allergies (Chiu et al., 2019). Serum and sputum inflammatory cytokines may be used as markers between bronchial asthma and chronic obstructive pulmonary disease (COPD) (Bai et al., 2019). IL-4, IL-5, IL-9, and IL-13 were shown in increasing levels in COPD, patients while in asthmatic recipients, the levels of TNF-α, IL-1β and IL-6 were found considerably increased (Bai et al., 2019). Asthma seems also to be related with particular phenotypes (Kuruvilla et al., 2019). In elderly asthmatic patients serum IL-33 and IL-31 levels were found lower, and this may contribute to less Th2 phenotype and asthma severity (Ulambayar et al., 2018). Bronchoalveolar lavage (BAL) fluids in asthmatic recipients showed elevated levels of IL-1RA, IL-1*α*, IL-1*β*, IL-2R*α*, IL-5, IL-6, IL-7, IL-8, G-CSF, GRO*α* (CXCL1), MIP-1*β* (CCL4), MIG (CXCL9), RANTES (CCL5) and TRAIL, eosinophils and neutrophils (Hosoki et al., 2015). Overall, neutrophils and IL-8 in BAL fluids seem to be the unique inflammatory markers to distinguish between controlled and uncontrolled asthma and disease severity (Hosoki et al., 2015).

Over-presented in asthmatic individuals was *Malassezia* genus (Nguyen et al.; Delhaes et al., 2012; van Woerden et al., 2013). *Aspergillus penicillium* and *Cladosporium* were also abundant in asthmatic cases (van Woerden et al., 2013). Several environmental *Basidomycota* species such as *Psathyrella candolleana, Grifolasordulenta*, and *Termitomycesclypeatus* were revealed from asthmatic individuals (Nguyen et al.).

We stated the importance of the newborn colonization (Bezirtzoglou and Stavropoulou, 2011). It is emphasized that early gut colonization by beneficial organisms such as *Lactobacilli* and *Bifidobacteria* is protecting us against different types of diseases (Ismail et al., 2016; Salminen et al., 2016). Early and elevated colonization by the bacterial genus of *Clostridium, Bacteroides fragilis, Streptococcus* and fungal populations of *Saccharomyces* and *Pichia kudriavzenii* predispose to asthmatic conditions (Nguyen et al.), as well as low amounts of the beneficial *Bifidobacterium* (Nguyen et al.; Ismail et al., 2016).

Gut bacterial burden is under the control of multiple factors, such as nutrition, environment, hormonal status, ageing (Turnbaugh et al., 2007), and are referred as gut microbiome. The gut microbiome is composed of trillions of bacteria and plays an important role in health and disease (Turnbaugh et al., 2007). The notion that the lung is sterile is abandoned (Huffnagle, 2015) as new molecular techniques gave evidence of bacterial populations in the lung (Huffnagle, 2015). However, bacterial concentration in the lung is shown humble compared to the gut communities (Hilty et al., 2010). Nevertheless, this cannot be generalized to the fungi kingdom as commensal fungi showed a protective effect on both local and systemic immunity due to their fungal wall mannans (Abt and Artis, 2013). As known sepsis is a potentially life-threatening state as a response to an infection due to chemicals produced into the blood against an infection. There is evidence in animals and humans that intestinal microbiota provide bacteria to the lung as abundance of *Bacteroides* observed in the lung following sepsis (Dickson et al., 2016b).

Shifts in microbial profile and decrease in the lung microbiota diversity is observed in chronic obstructive pulmonary disease (COPD) (Huang et al., 2010). The 16S rRNA PhyloChip analysis makes known that more than 1,200 bacterial taxa belonging to 140 distinct families were detected in the airways (Huang et al., 2010). The phylum *Proteobacteria* including families of *Pseudomonadaceae, Enterobacteriaceae*, and *Helicobacteraceae* was predominant (Wang et al., 2016). In intubated patients. *Haemophilus influenzae* and *Pseudomonas aeruginosa* were also found in COPD exacerbations (Huang et al., 2010). Moreover, rise in intestinal permeability and release of adrenal hormone metabolites were linked to short or long term mortality in COPD patients (Zurfluh et al., 2018).

A decrease in the lung microbiota diversity is observed in cystic fibrosis patients (Cox et al., 2010). Diversification was more pronounced in younger individuals than older ones with cystic fibrosis. Bacterial populations involved are members of *Pseudomonadaceae, Xanthomonadaceae, Moraxellaceae* and *Enterobacteriaceae* (Cox et al., 2010). The genus *Streptococcus, Prevotella, Rothia, Veillonella, Acintomyces, Neisseria, Haemophilus, Gemellaare* were isolated in cystic fibrosis during exacerbations in pediatric patients (Worlitzsch et al., 2009). However, *Pseudomonas aeruginosa* and *Staphylococcus aureus* are incriminated in most cystic fibrosis cases (Worlitzsch et al., 2009). *Streptococcus, Prevotella, Rothia, Veillonella, Acinetomyces, Pseudomonas* are more frequently involved in cystic fibrosis adult cases (Delhaes et al., 2012). Over-presented in cystic fibrosis were *Aspergillus penicillium, Aspergillus fumigatus, Malassezia*, *Candida albicans* and *Candida parapsilosis* (van Woerden et al., 2013). *Cladosporium cladosporiodes* has been isolated in immunocompromised patients and *Malassezia pachydermatitis* was also found in immunocompromised hosts and in atopic dermatitis (Nguyen et al.). *Aspergillus fumigatus* was associated with corticoid treatment (Fraczek et al., 2019). The authors stated a shift in bacterial and fungal population dominated by *Proteobacteria* phylum which includes many Gram-negative bacteria (Dickson et al., 2016b).

## The Lung and COVID-19

Our specific interest is aroused by the novel beta-coronavirus SARS-CoV-2 affecting seriously the respiratory system and causing an acute respiratory disease known as COVID-19. SARS-CoV-2 has recently taken over our attention due to the COVID-19 pandemic because of its contagiousness as well as unexpected mortality rates. SARS-CoV-2 belongs to the Coronaviridae which is a family of enveloped, positive-sense, single-stranded RNA (+ssRNA) viruses. SARS-CoV-2 contains four basic structural glycoproteins: spike (S), membrane (M), envelope (E), and nucleocapsid (N) (de Wit et al., 2016).

As little is known about the infection process of this novel virus, we turned our particular attention to this pathogen. As of November 2020, the number of COVID-19 confirmed cases exceeded 64 millions of cases with more than 1,500,000 deaths. The large majority of diseased seems to develop a mild disease or even being asymptomatic as they mount a suitable immune response. Nevertheless, several patients develop severe clinical images, and this is linked to the insufficient response of their immune system and underlying pathologies. In fact, the mechanisms involved in the progression of the disease remains yet obscure.

As stated previously, SARS-CoV-2 contains four structural glycoproteins. Among them the envelop glycoprotein E, the nucleocapsid protein N, and the membrane glycoprotein M are responsible for viral assemblage and binding to the host cells (Li, 2012).

The human angiotensin converting enzyme 2 (ACE2) was identified as the receptor for SARS-CoV-2 virus which permits access to the endocytic uptake (Petersen et al., 2020). This enzyme is expressed on the human lungs’ epithelium and catalyzes the conversion of angiotensin II (Letko et al., 2020; Ou et al., 2020). ACE 2 can be found in a wide variety of human tissues, such as kidney, intestine, heart, thyroid, adipose tissue and testis where viremia could happened in case of contamination (Li M. et al., 2020). Neurological symptoms are also reported due to CNS affection (Abboud et al., 2020).

The role of the spike glycoprotein S acts in connection with angiotensin. Specifically, this glycoprotein is cleaved (Anand et al., 2020) by a cellular derived protease into two glucoproteins S1 and S2. The glycoprotein S1 binds to the angiotensin, while S2 is activated by the host TMPRSS2 (Transmembrane Serine Protease) resulting in a membrane fusion which permits the virion to enter the host cells *via* receptor-mediated endocytosis (Petersen et al., 2020). In contrast to the majority of coronaviruses, SARS-CoV-2 expresses a furin-like protease. It is believed that this furin-like protease may contribute to the widened cell tropism and enhanced transmissibility of the virus (Shapiro et al., 1997). Moreover, it was shown that a Two-Pore Channel (TPC2) is crucial for the entry of SARS-CoV-2 into host cells (Ou et al., 2020). The Two-Pore Channels (TPCs) are entry channels into the endo-lysosomal system. In fact, the intracellular messenger nicotinic acid adenine dinucleotide phosphate (NAADP) mobilizes calcium from acidic organelles through two-pore channels (Calcraft et al., 2009). Moreover, the authors demonstrated that S-protein *via* the Transmembrane Serine Protease 2 (TMPRSS2) facilitates SARS-CoV-2 entry into host cells (Baughn et al., 2020; Hoffmann et al., 2020).

Following attachment to the ACE 2 receptor, there is membrane and viral fusion with the aid of glucoprotein S as stated previously (Petersen et al., 2020) which permits SARS-CoV-2 virus entry into the cells (Masters, 2006). After the virus enters the host cells, viral material is replicated by the aid of RNA polymerase, and another viral RNA released enzymes (Ratia et al., 2006). As known viral genome encodes the replicases PP1a and PP1ab which are cleaved by the 3CLPro (3C-Like Protease)and the PLPro (Parpain-Like Protease) into 16 nonstructural proteins as RNA dependent RNA polymerases (RdRp) shaping the replication complex (Ratia et al., 2006). During genome translation coronavirus replication induces ribosome frameshifting (Masters, 2006) before reassembly, encapsulation and exocytosis of the mature virions out of the host cell able to infect new host cells.

SARS-CoV-2 as an antigen exposed to the host Antigen Presenting Cells (APCs) produces releasing of inflammatory mediator cytokines such as interleukin-1 (IL-1), interleukin-6 (IL-6), CXCL-10 and tumor necrosis factor alpha (TNF-alpha). These cytokines are producing an excessive pro-inflammatory response called cytokine storm to the host which damages lung epithelium (Sanghai and Tranmer, 2020). Moreover, pathogens induce a pro-inflammatory response in epithelial cells by activating the transcription Nuclear Factor-*κ*B (NF-*k*B) which regulates innate and adaptive immune functions, inflammation, and cell proliferation (Liu et al., 2017). It is of note that SARS-CoV-2 is a cytopathic virus which damages directly the alveolar epithelium in the lungs and induces epithelial cell death. This damage may occur to multiple organs causing a multi-organ failure of the host (Xu D. et al., 2020).

As it is known the expression of Pattern Recognition Receptors (PRRs) is enhanced in the lung cells during inflammation. As a response, macrophages, monocytes, and neutrophils increase levels of PAMPs (Pathogen-Associated Molecular patterns) and DAMPs (Danger-Associated Molecular Patterns) (Mogensen, 2009). PAMPs are nucleic acids or glycoproteins recognized by PRRs and expressing cytokines and other co-stimulatory components against the pathogenic virus which activate as well then antigen presenting cells and the specific adaptive immunity (Mogensen, 2009). DAMPs are found intracellularly and participates in the activation of the inflammasome as well as the conversion of proIL-1 to activeIL-1 (Law et al., 2009). Notably, the TLRs (Toll Like Receptors) membrane glycoproteins are the most known PRRs (Law et al., 2009).

On the other side, traditional biomarkers of acute infection such as C-Reactive Protein, ferritin, neutrophils, and leucocytes must be considered. Recent evidence stated that neutrophils and complement are involved in a maladaptive immune response as complement interacts with the platelet/neutrophil extracellular traps (NETs)/thrombin axis which leads to enhanced inflammation, ombotic microangiopathies and high mortality (Skendros et al., 2020). Similarly, the unbalance of the immune system due to the excessive cytokines releasing in response to SARS-CoV-2 invasion leads to an abnormal hypercoagulation with thrombotic events (Jose and Manuel, 2020).

Researchers argued the importance of the gut microbiota in relation to the development of immunity against SARS-CoV-2 and the recovery impact (Xu K. et al., 2020). Intestinal dysbiosis, with a decrease in the beneficial probiotic microbiota, is observed in subjects with COVID-19 (Xu K. et al., 2020).

As stated previously, the gut microbiome impact upon Influenzae virus (IV) and Respiratory Syncytial Virus (RSV) (Groves et al., 2018). Shifts in gut microbiome of COVID-19 patients were effectively (Zuo et al., 2020) characterized by depletion of the beneficial microbiota and accrued levels of opportunistic pathogens. Putrefactive bacteria such as several *Clostridium species (C. ramosum, C.hathewayi)* and *Coprobacillus* were present in abundance and correlated to the disease severity (Zuo et al., 2020). In contrast, the beneficial commensals *Faecalibacterium prausnitzii*, *Eubacterium ventriosum Roseburia*, *Lachnospiraceae*, *Alistipes onderdonkii* and *Bacteroides ovatus* were found in low levels in COVID-19 patients (Zuo et al., 2020). Patients fecal specimens showed an inverse correlation between SARS-CoV-2 levels and the presence of *Bacteroides* sp which are involved in downregulation expression of the angiotensin-converting enzyme 2(ACE2) (Zuo et al., 2020). ACE-2 receptors are the entry point into cells for SARS-CoV-2. It is of note that they are expressed in few distinct anatomical seats comprising the gut and the lungs.

The authors stated the accrued levels of *Streptococcus* in patients’ fecal microbiota to be correlated with infection risk by opportunistic pathogenic bacteria (Khatiwada and Subedi, 2020), such as *Rothia, Veillonella*, and *Actinomyces* (Khatiwada and Subedi, 2020).Moreover, opportunistic fungal pathogens such as *Candida albicans, Candida auris, and Aspergillus flavus* are detected in patients’ microbiome (Khatiwada and Subedi, 2020). The authors stated the value of the gut microbiota in COVID-19 patients as a dynamic diagnostic biomarker and therapeutic tool (Gu et al., 2020).

Other studies of bronchoalveolar lavage fluid, lung *post-mortem* biopsies and gut microbiota from COVID-19 patients showed important shifts in the gut microbiota balance as a mixed bacterial/fungal infection occurred (Fan et al., 2020).

Increased lung loading with gut bacteria seems to be a predictive marker to the acute respiratory distress syndrome (ARDS) malefic consequences as dysbiosis is taking place dysregulating the immune response and leading to inflammation (Yang et al., 2020). The composition of the gut microbiome may be used as a predictive tool of the disease development and infection severity (Chu and Mazmanian, 2013). Based on the above, scientists developed a proteomic risk score’ (PRS) acting as a predictive tool using machine learning algorithms. Putrefactive bacteria such as *Ruminococcus* and *Clostridium* spp were found under dysbiosis in high numbers while beneficial probiotic bacteria such as Lactobacillus and *Bifidobacterium* were absent (Gou et al., 2020).

## Conclusions and Remarks

Further research focusing on the intestinal–respiratory microbiome interconnection is likely to reveal important aspects into the dynamics of the microbiomes. Getting an in-depth understanding on the role of intestinal dysbiosis may elucidate the pathogenesis of different diseases. Notably, it is crucial to bring more light in the interconnecting reciprocal dialogue between the lung and the gut as its microbial kingdom keeps a significant impact in this interconnection which needs further investigation. Without any doubt, new technologies including high-throughput and genome sequencing methodologies will enrich our knowledge about the role of the gut and lung microbiomes in the development of the respiratory diseases. Getting knowledge of the involved physio-pathological mechanisms will help the medical community to find solutions for the treatment of COVID-19 (Stavropoulou and Bezirtzoglou, 2020).

Medicines inactivating the enzymes involved in these mechanisms should be promising as potential antiviral treatments (Sze et al., 2014). Moreover, considering the role and the importance of the microbiota dynamics and homeostasis, the probiotic approach in the treatment of the respiratory disease seems to keep an important impact as prophylactic or therapeutic agents, especially when the failure of antibiotics occurs.

At this end, we conclude that more basic and clinical studies should be done in order to clarify the role of the physio-pathological mechanisms and human organs and systems dialogues in health and disease.

## Author Contributions

Conceptualization, original draft preparation, and editing, ES. Formal analysis, TK and KK. Investigation, ES. Resources, CV. Writing—original draft preparation and editing, CT. Editing, ThK. Supervision, original draft preparation, and editing, EB. All authors contributed to the article and approved the submitted version.

## Conflict of Interest

The authors declare that the research was conducted in the absence of any commercial or financial relationships that could be construed as a potential conflict of interest.
